# Minimally Invasive versus Open Liver Resection for Stage I/II Hepatocellular Carcinoma

**DOI:** 10.3390/cancers13194800

**Published:** 2021-09-25

**Authors:** Emrullah Birgin, Sarah R. Kaslow, Svetlana Hetjens, Camilo Correa-Gallego, Nuh N. Rahbari

**Affiliations:** 1 Department of Surgery, Universitätsmedizin Mannheim, Medical Faculty Mannheim, Heidelberg University, 68167 Mannheim, Germany; emrullah.birgin@umm.de; 2 Department of Surgery, NYU Grossman School of Medicine, New York, NY 10016, USA; sarah.kaslow@nyulangone.org; 3 Department of Medical Statistics and Biomathematics, Medical Faculty Mannheim, Heidelberg University, 68167 Mannheim, Germany; svetlana.hetjens@medma.uni-heidelberg.de

**Keywords:** laparoscopic surgery, robotic surgery, hepatectomy, liver cancer, survival

## Abstract

**Simple Summary:**

The type of surgical approach for the treatment of hepatocellular carcinoma is unclear. This study compared minimally invasive to open liver resections using the National Cancer Database. The results showed a similar overall survival with improved perioperative outcomes, but higher rates of positive resection margins in patients with minimally invasive liver resections. The higher rate of residual tumors requires further investigation.

**Abstract:**

Minimally invasive liver resection (MILR) is increasingly used as a surgical treatment for patients with hepatocellular carcinoma (HCC). However, there is no large scale data to compare the effectiveness of MILR in comparison to open liver resection (OLR). We identified patients with stage I or II HCC from the National Cancer Database using propensity score matching techniques. Overall, 1931 (66%) and 995 (34%) patients underwent OLR or MILR between 2010 and 2015. After propensity matching, 5-year OS was similar in the MILR and OLR group (51.7% vs. 52.8%, *p* = 0.766). MILR was associated with lower 90-day mortality (5% vs. 7%, *p* = 0.041) and shorter length of stay (4 days vs. 5 days, *p* < 0.001), but higher rates of positive margins (6% vs. 4%, *p* = 0.001). An operation at an academic institution was identified as an independent preventive factor for a positive resection margin (OR 0.64: 95% CI 0.43–0.97) and 90-day mortality (OR 0.61; 95% CI 0.41–0.91). MILR for HCC is associated with similar overall survival to OLR, with the benefit of improved short term postoperative outcomes. The increased rate of positive margins after MILR requires further investigation, as do the differences in perioperative outcomes between academic and nonacademic institutions.

## 1. Introduction

Hepatocellular carcinoma (HCC) is the most common primary liver tumor, with an increasing incidence and mortality worldwide [1]. Even though several new agents have been introduced for the first and second line treatment of patients with advanced disease, there has been little change in the management of patients with stage I/II tumors [2]. For patients with localized HCC, surgical therapy remains the primary treatment, with the prospect of cure in 30–50% of cases [3]. Approximately 80% of HCC patients have underlying liver cirrhosis [4]. Although liver transplantation is the preferred treatment for HCC in the setting of cirrhosis, this treatment can only be offered to a fraction of patients due to organ shortage and strict selection criteria that are frequently not met [5]. Therefore, the role of surgical resection in the management of localized HCC has increased in recent years [6]. This is mirrored by changes in clinical algorithms for HCC treatment [7]. In the past, recommendation for surgical resection was limited to patients with single nodules of ≤3 cm. However, in updated guidelines, Grade A recommendation is given for surgical resection as primary treatment for patients with a solitary tumor of any size, or up to three nodules of ≤3 cm (Barcelona Clinic Liver Cancer (BCLC) stage 0/A), and as an alternative treatment for patients with multinodular disease (BCLC stage B) [8]. Minimally invasive liver resection (MILR) was introduced in the early 1990s as a surgical approach for patients with benign lesions in peripheral locations of the liver [9]. Thereafter, advances in surgical technology and imaging have broadened the indications for MILR to patients with malignant liver lesions, including those requiring major hepatectomies. The indications for and implementation of MILR have been summarized in international consensus statements [10]. In the most recent consensus statement, MILR has been promoted as advantageous in terms of blood loss, hospital stay and posthepatectomy liver failure (PHLF) risk, and comparable to open liver resection (OLR) with respect to operating time, resection margins, and recurrence rates [11]. In the absence of data from large scale prospective randomized trials, these statements were based on single-center studies and meta-analyses of cohort studies [12]. However, neither randomized trials nor cohort studies from specialized institutions reflect the actual reality of surgical care and outcomes on a large scale, across multiple different institutions with varying levels of specialization. Therefore, we carried out an observational study on the effectiveness and safety of MILR vs. OLR for the management of stage I/II HCC using the National Cancer Database (NCDB).

## 2. Materials and Methods

Patients with HCC were identified in the NCDB. The NCDB is a national cancer registry capturing approximately 70% of all newly diagnosed cancer cases, annually, in more than 1500 accredited hospitals in the United States [13]. By using completely de-identified data, the local institutional review board approved the current study as exempt human research.

**Figure 1:**
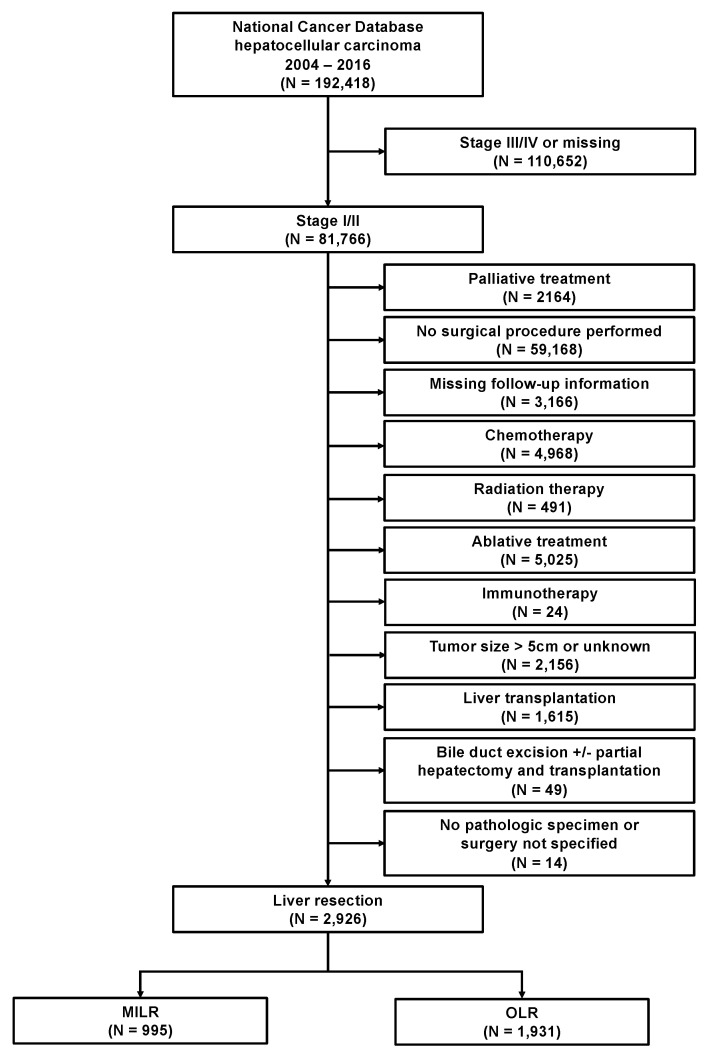
Flow diagram. AJCC, American Joint Committee on Cancer; MILR, minimally invasive liver resection; OLR, open liver resection.

A total of 192,418 patients with an HCC diagnosis between 2004 and 2016 were assessed for eligibility. Figure 1 shows the study cohort in line with the STROBE (Strengthening the Reporting of Observational Studies in Epidemiology) guideline [14]. We included patients with a clinical stage I and stage II diagnosis who underwent a curative intent liver resection as primary treatment. Clinical stage included clinical T, N, and M elements, as defined by the American Joint Committee on Cancer (AJCC). The 7th edition of the AJCC was used for the definition of clinical stages I and II, including a tumor size of <5 cm. Patients with missing follow up information, palliative treatments, or other types of nonsurgical treatment modalities, including chemotherapy, radiation therapy, ablative treatments, and immunotherapy, were excluded. Patients who underwent liver transplantation, not specified surgical procedures, had missing pathologic specimen, or underwent primarily bile duct resections with or without a partial hepatectomy/liver transplantation, were further excluded. Given that the type of surgical approach was first documented in the NCDB in 2010, patients diagnosed with HCCs before 2010 were further excluded. The final study population included 2926 patients with available treatment information on the specified surgical approach. We gathered demographic characteristics, including patient age at diagnosis, gender, race, year of diagnosis, treating facility, the distance between residence and facility, educational attainment (high school degree), residence area, inferred annual household income, and insurance status. Clinical characteristics included comorbidities (Charlson/Deyo score), clinical stage, tumor size, TNM-stage, grading, lymph-vascular invasion, and resection margin. We retrieved data on postoperative outcomes, such as 30-day unplanned readmissions, 30-day-mortality rates, 90-day mortality rates, and overall survival data. We included data on the type of liver resection and the surgical approach (robotic, laparoscopic, open). Minimally invasive liver surgery was defined as robotic and/or laparoscopic liver surgery.

The primary study outcome was to compare the overall survival, measured from the date of cancer diagnosis to death of any cause or last contact, in patients with clinical stage I or II HCC treated by minimally invasive liver resection (MILR) or by open liver resection (OLR). Secondary objectives included early perioperative outcomes and to determine factors associated with the utilization of minimally invasive liver resections for HCC.

The conditional probability of having received MILR was estimated by generating propensity scores using multivariate logistic regression. The following covariates were included in the model: age, gender, race, year of diagnosis, facility type, the distance between residence and facility, income, insurance type, residence area, high school degree, comorbidity, clinical TNM stages, and clinical stage group. In line with previous reports, only variables collected before therapy initiating were used in the model, and the pathologic stage was not included [15,16]. Patients with MILR were matched 1:1 to patients treated by OLR on propensity score using the nearest neighbor algorithm with a caliper width equal to 0.1 standard deviations (without replacement) [17]. The balance of covariates was analyzed by using standardized differences between groups and a standardized difference below 0.1 was considered as an indicator of balance.

Categorical data was assessed by using Pearson χ^2^ or Fisher’s exact test and continuous data was analyzed using the Wilcoxon rank sum test or student’s t-test. Kaplan–Meier estimators were calculated for the primary outcome and compared using the stratified log-rank test for each treatment group, as well as the subgroups clinical stage and resection margin. The association of perioperative variables and overall survival was analyzed using a multivariable Cox proportional hazards regression model, adjusted for patient and facility factors. Subgroup analyses were performed within the matched study group for selective variables to explore the heterogeneity of treatment effects using tests of interaction. Multivariable logistic regression analyses were performed for clinical factors associated with positive resection margin and 90-day mortality, including variables with a *p* value ≤ 0.100 on univariable analyses. All statistical analyses and propensity matching were performed with R version 4.0.3 (Vienna, Austria). Graphpad was used for data visualization.

## 3. Results

### 3.1. Use of MILR as a Surgical Approach for HCC

**Figure 2:**
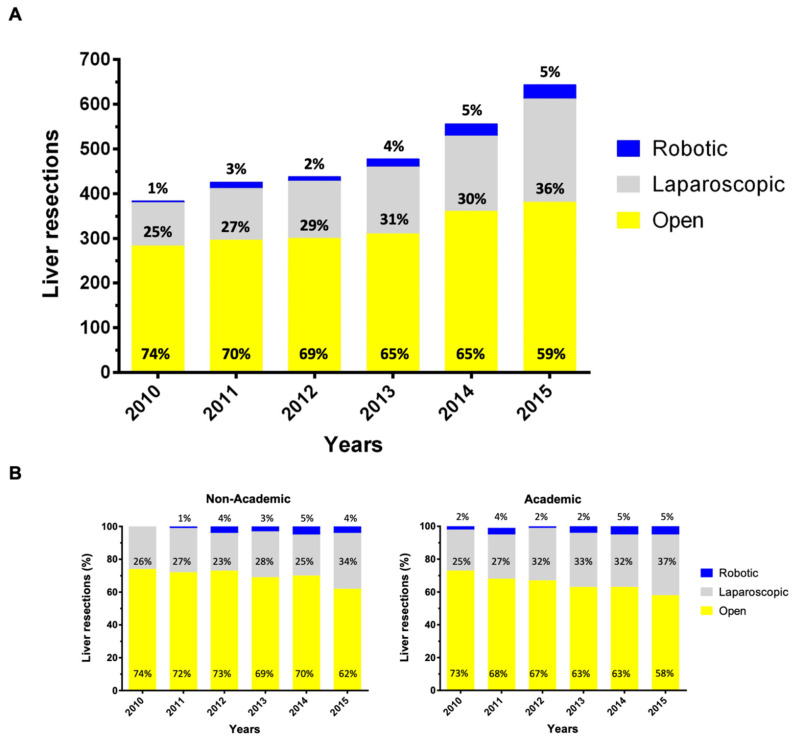
Trends of minimally invasive liver resection utilization for stage I/II HCC treatment. Use of minimally invasive surgery (laparoscopic and robotic) versus open hepatectomy for stage I or II hepatocellular carcinoma over time in the unmatched study population from the National Cancer Center Database 2010-2015 (**A**) and stratified by the facility type (**B**).

Applying strict eligibility criteria, a total of 2926 patients were identified who underwent liver resection as primary treatment for stage I/II HCC between 2010 and 2015; MILR (*n* = 995; 34%) or OLR (*n* = 1931; 65%) (Figure 1). During the study period, the annual number of liver resections for HCC steadily increased from 383 in 2010 to 644 in 2015. Along with this development, the proportion of patients who received MILR has also increased significantly between 2010 and 2015 (*p* < 0.001). While a laparoscopic or robotic approach was utilized in 25% and 1%, respectively, of all resections in 2010, 36% and 5% were so in 2015 (Figure 2A). Stratified analyses for the facility type revealed this trend as being primarily caused by the increased use of MILR at academic institutions (Figure 2B).

Patients’ clinicopathological characteristics are summarized in Table 1. In the unmatched cohort, patients in the MILR group were more frequently treated at an academic institution (*p* = 0.015) and at an East North Central region (*p* < 0.001) and less frequently underwent an (extended) lobectomy (*p* < 0.001). After propensity score matching, both groups were well balanced for all baseline/clinicopathologic covariates, including the type of surgical procedure (Figure S1). 

### 3.2. Histopathological Results and Perioperative Outcome after MILR vs. OLR for Stage I/II HCC

The results of perioperative outcomes are reported in Table 2. The comparisons of the histopathological results revealed similar findings between both groups for tumor grading, presence of lymph-vascular invasion, and T stage, whereas there was a higher proportion of patients in whom the lymph node status could not be assessed (Nx) in the OLR group (*p* < 0.001). However, this result is likely to reflect the lack of standardized lymphadenectomy, as almost all patients with available nodal status had negative lymph nodes and lymph node status was not associated with overall survival. Importantly, we found a significantly higher rate of positive resection margins after MILR vs. OLR (6.9% vs. 3.8%; *p* = 0.006), whereas the rate of unknown resection margins was similarly low in the MILR and OLR groups (3.5% vs. 3.0%). To gain insight into which factors predisposed to a positive resection margin, further analyses were carried out. These analyses confirmed MILR as an independent risk factor for margin positivity (Odds ratio [OR] 1.89: 95% confidence interval [CI] 1.25–2.85; *p* = 0.002). Furthermore, an operation at an academic institution was found to be inversely associated with a positive resection margin (OR 0.61; 95% CI 0.40–0.94; *p* = 0.025 (Table S1).

The median hospital stay was lower in patients with MILR (4 vs. 5 days; *p* < 0.001). The unplanned readmission rate and 30-day mortality rates were higher in the OLR group, though these differences failed to reach statistical significance. However, we found a significantly higher 90-day mortality in patients who underwent OLR (4.5% vs. 6.7%; *p* = 0.04). Further analyses confirmed an MILR as an independent preventive factor for 90-day mortality (OR 0.65; 95% CI 0.44–0.96; *p* = 0.031). In addition to a lobectomy (OR 3.12; 95% CI 2.04–4.80; *p* < 0.001), age 50-59 (OR 3.52; 95%CI 1.04–11.90; *p* = 0.043), a comorbidity index of 3 (OR 1.98; 95% CI 1.11–3.52; *p* = 0.020), year of diagnosis in 2012 (OR 0.33; 95%CI 0.14–0.77; *p* = 0.010), were revealed as independent predictors of 90-day mortality, whereas a protective effect was found for an operation at an academic institution (OR 0.61; 95% CI 0.41–0.91; *p* = 0.015) (Table S2).

### 3.3. Survival Analysis of MILR vs. OLR for Stage I/II HCC

**Figure 3:**
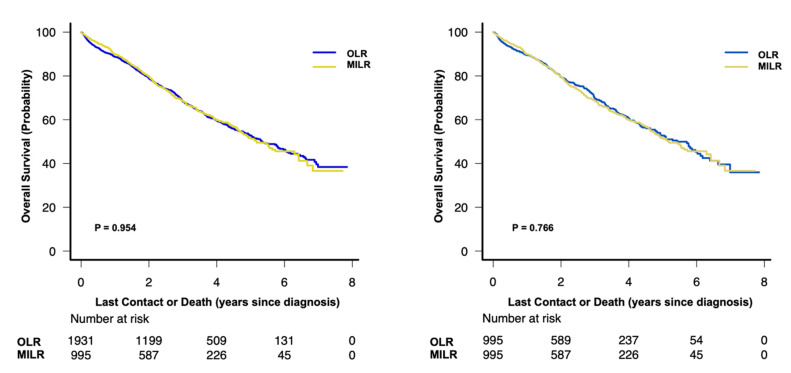
Overall survival in (A) the unmatched and (B) the propensity score matched study cohort with stage I or II hepatocellular carcinoma (log-rank test). MILR, minimally invasive liver resection; OLR, open liver resection.

Patients in both groups were followed for a median duration of 30 months (interquartile range, 16 to 48 months). During the study period, we observed 1018 and 657 deaths in the unmatched and propensity score matched cohorts, respectively, which translates into a 5-year overall survival rate of 52.5% (95% CI 50.0%–54.9%) and 52.2% (95% CI 49.0%–55.3%). In the unadjusted analyses there was no difference in the 5-year overall survival rate between patients with MILR and OLR for the unmatched cohort (51.7%, 95%CI 47.1–56.1% vs. 52.8%, 95%CI 49.8–55.8%; *p* = 0.954) or the propensity score matched cohort (51.7%, 95%CI 47.1–56.1% vs. 52.8%, 95%CI 48.2–57.1%; *p* = 0.766) (Figure 3). 

**Figure 4:**
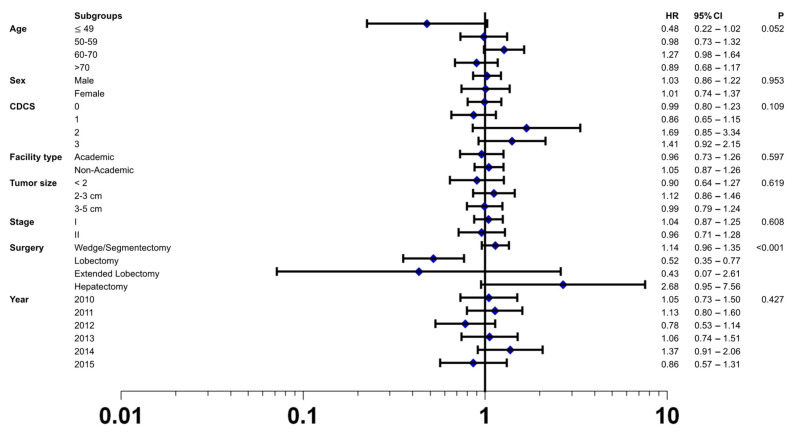
Forest plot depicting hazard ratio of minimally invasive liver resection versus open liver resection in the matched study cohort using the tests of interaction in the following subgroups: age, sex, CDCS (Charlson Deyo Comorbidity Index Score), facility type, tumor size, stage, surgery, and year. The open liver resection group was used as reference. Black squares represent hazard ratios (and 95% confidence intervals by the corresponding horizontal lines).

This finding of a comparable overall survival after MILR vs. OLR was confirmed in the multivariable Cox regression analyses of the matched cohort (HR 1.02; 95% CI 0.88–1.20; *p* = 0.763). In addition, this analysis confirmed known prognostic factors for patients with localized HCC undergoing surgical resection, such as clinical tumor stage, tumor size, tumor differentiation, presence of lymph-vascular invasion and positive resection margin (Table S3). To further explore the possible treatment effects of the surgical approach on postoperative overall survival, a priori defined subgroup analyses were carried out. These analyses demonstrated a consistent effect of MILR over OLR, except for the type of surgical procedure as indicated by the forest plot analyses in Figure 4. Patients who underwent a lobectomy in a minimally invasive fashion had a survival benefit compared to OLR. Furthermore, there was a trend of a survival benefit in younger patients (≤49 years) who received MILR compared to OLR.

## 4. Discussion

Even though surgical resection remains the primary treatment in patients with localized HCC, no consensus has been reached as to which surgical approach yields superior short- and long-term outcomes [18]. To compare the effectiveness of MILR vs. OLR and gain insight into current clinical practice and outcomes, we conducted an analysis of patients included in the NCDB with a rigorous methodological approach. In the absence of multicenter randomized trials, we applied propensity score matched analysis to obtain well balanced groups with respect to all relevant clinicopathologic covariates. Our results demonstrate that the proportion of patients receiving MILR has been increasing over time, yet most operations remain to be carried out as OLR. Patients who undergo MILR have a significantly shorter hospital stay and a 33% lower risk of death at 90 days after surgery. Importantly, we found a significantly higher rate of positive resection margins in patients with MILR. Despite this finding and a significant and independent association of positive resection margins with overall survival, our comparison of long-term outcomes revealed similar overall survival between both groups.

Minimally invasive surgery (MIS) has been established for the resection of various gastrointestinal malignancies. For liver malignancies, multicenter randomized data is available from the COMET trial in which laparoscopic and open surgery were compared in 280 patients with colorectal liver metastases [19]. This trial demonstrated lower postoperative complications and hospital stays for patients in the laparoscopic surgery arm, whereas there was no significant difference in intraoperative blood loss, operating time, and perioperative mortality between both groups. The results of our analysis of patients with HCC confirm the benefit of MILR for patients’ perioperative recovery, as reflected by a significantly shorter hospital stay. Together with the findings of numerous other studies that demonstrated lower pain, earlier mobilization, and more rapid return of gastrointestinal function after minimally invasive compared to open surgery for various abdominal malignancies, these data extend the benefits of MILR on postoperative recovery to patients with HCC.

About 80–90% of HCC arise in the background of advanced fibrosis/cirrhosis because of chronic parenchymal damage of the liver. It is well known that patients with chronic liver disease are more prone to various surgical stress conditions, such as hemorrhage, trauma, and infection [20]. Although the molecular mechanisms of the body’s stress response to the surgical insult are not fully understood yet, there is evidence that their extent and consequences depend on the magnitude of the insult. In line with this observation, the surgical stress response is attenuated in patients undergoing minimally invasive, as opposed to open, surgery [21]. In a patient with normal or mildly impaired liver function, the more pronounced surgical stress response after open surgery may explain the delayed recovery and increased perioperative complications rates. However, in patients with more advanced chronic liver disease, the consequences might be more significant, and potentially fatal. Our results revealed a higher unplanned readmission rate and significantly higher 90-day mortality in patients undergoing OLR. Although details of patients underlying liver function were not available for analyses, nor were data on patients’ perioperative complications, these findings favor the notion that, in patients with chronic liver disease, the trauma caused by the surgical approach contributes to the perioperative risk. Further support for a particularly beneficial role of MIS in the setting of chronic liver disease can be obtained from studies that demonstrated acceptable perioperative outcomes for the resection of HCC in patients with advanced cirrhosis (i.e., Child B and or clinically relevant portal hypertension) [22,23,24]. While, historically, these conditions were considered contraindications to resection, these emerging data on MILR have changed clinical decision making and positioned liver resection as a primary or alternative treatment option in current guidelines [25]. 

It is an important finding of our study that MILR of stage I/II HCC was independently associated with a significantly increased risk of positive resection margins. This is an unexpected finding, particularly for the current analysis of stage I/II tumors, and differs from data on margin clearance in patients undergoing MILR vs. OLR for colorectal liver metastases [19]. There might be several explanations for this finding. Liver resection in a diseased liver is technically more demanding due to the more difficult transection of the parenchyma, bleeding diathesis, and impaired visualization of the tumor on intraoperative ultrasound. One might speculate that these factors were more relevant for obtaining clear margins in MILR. In fact, it has been shown that a minimum of 55 resections is required for surgeons to pass the learning curve in MILR [26]. However, data on surgeons’ expertise was not available for this analysis. As an alternative explanation, the presence of chronic liver disease with impaired liver function might have prompted surgeons to opt for narrower margins to save nontumorous liver parenchyma. While the minimum required margin width in HCC remains a matter of ongoing debate, laparoscopic assessment of the margin distance is more challenging and might have resulted in a higher incidence of positive margins. In addition, tumor detachment from major intrahepatic vessels in HCC has recently been introduced to be oncologically adequate, with comparable survival and recurrence rates to R0 resections [27]. Unfortunately, the dataset provided neither data on the location of margin positivity, nor tumor location. While exophytic or pedunculated HCCs bear a higher risk of tumor rupture affecting both resection margins and prognosis, the incidence remains particularly rare in Western cohorts [28,29].

Our analyses revealed an operation at an academic institution being inversely associated with positive resection margins. Together with the observed lower 90-day mortality, these data further support the centralization of surgical care for stage I/II HCC.

There is limited data on the effects of MILR on long term survival after resection of HCC. The available studies showed comparable long-term survival for patients with MILR vs. OLR for HCC [22,30]. Our analyses confirmed these data and showed similar overall survival for patients in both groups, which remained consistent in subgroups analysis of clinically relevant variables. Recently, intriguing data were reported from an individual patient data meta-analysis on long term survival after resection of colorectal liver metastases, with a survival benefit in patients who underwent MILR [31]. Although our data do not show a survival advantage for patients with MILR vs. OLR for stage I/II HCC, the higher rate of margin positivity with its independent prognostic value needs to be appreciated.

There are some limitations to the present study. In the dataset, there was data lacking on liver function parameters, AFP level, tumor location (i.e., perivascular, exophytic), details of surgical techniques, surgeons’ experience level, details of complications, and cause of death. Therefore, these covariates could not be assessed for the propensity score matching. Furthermore, we included all patients between 2004 and 2016 in the dataset, however, the final cohort comprised only patients operated on between 2010 and 2015, due to our strict eligibility criteria. Although the present study reflects the largest cohort study in literature on MILR versus OLR for HCC, to the best of our knowledge, these limitations might still have caused selection bias despite our rigorous statistical methodology.

## 5. Conclusions

The present study of a large dataset revealed MILR to be safe and advantageous with respect to perioperative outcome after resection of stage I/II HCC. Although there was no difference in overall survival between both groups, the significantly higher rate of positive resection margins in the MILR group is concerning and requires further investigation.

## Figures and Tables

**Table 1:** Demographic and Clinical Characteristics of Patients with Hepatocellular Carcinoma Before and After Propensity Score Matching.

Characteristics	MILR	Unmatched OLR		Matched OLR	
	*n* = 995	*n* = 1931	*p* ^1^	*n* = 995	*p* ^2^
Age, years			0.864		0.626
≤49	62 (6.3)	124 (6.4)		65 (6.5)	
50–59	252 (25.3)	512 (26.5)		272 (27.3)	
60–70	426 (42.8)	822 (42.6)		399 (40.1)	
≥71	255 (25.6)	473 (24.5)		259 (26.1)	
Sex			0.052		0.805
Male	701 (70.5)	1427 (73.9)		707 (71.1)	
Female	294 (29.5)	504 (26.1)		288 (28.9)	
Race			0.169		0.784
White	827 (83.1)	1550 (80.3)		822 (82.6)	
Black	155 (15.6)	354 (18.3)		156 (15.7)	
Other/Unknown	13 (1.3)	27 (1.4)		17 (1.7)	
Comorbidity Index			0.076		0.570
0	545 (54.8)	1099 (56.9)		560 (56.3)	
1	285 (28.6)	500 (25.9)		273 (27.4)	
2	68 (6.8)	170 (8.8)		56 (5.6)	
3	97 (9.8)	162 (8.4)		106 (10.7)	
Year of Diagnosis			<0.001		0.954
2010	101 (10.1)	283 (14.7)		100 (10.0)	
2011	130 (13.1)	296 (15.3)		144 (14.5)	
2012	138 (13.9)	300 (15.5)		140 (14.1)	
2013	168 (16.9)	310 (16.1)		169 (17.0)	
2014	195 (19.6)	361 (18.7)		184 (18.5)	
2015	263 (26.4)	381 (19.7)		258 (25.9)	
Facility Type			0.015		0.885
Community	12 (1.2)	24 (1.2)		8 (0.8)	
Comprehensive Community	136 (13.6)	355 (18.4)		128 (12.9)	
Academic/research	725 (72.9)	1313 (68.0)		735 (73.9)	
Integrated network	109 (11.0)	203 (10.5)		112 (11.2)	
Missing	13 (1.3)	36 (1.9)		12 (1.2)	
Facility Location			<0.001		0.153
New England	70 (7.0)	116 (6.0)		61 (6.1)	
Middle Atlantic	206 (20.7)	408 (21.1)		212 (21.3)	
South Atlantic	182 (18.3)	365 (18.9)		178 (17.9)	
East North Central	193 (19.4)	237 (12.3)		137 (13.8)	
East South Central	54 (5.4)	117 (6.1)		55 (5.5)	
West North Central	56 (5.6)	134 (6.9)		79 (7.9)	
West South Central	83 (8.4)	201 (10.4)		106 (10.7)	
Mountain	23 (2.3)	57 (3.0)		36 (3.6)	
Pacific	115 (11.6)	260 (13.5)		119 (12.0)	
Missing	13 (1.3)	36 (1.8)		12 (1.2)	
Distance to Facility			0.215		0.972
<12.5 miles	466 (46.8)	929 (48.1)		464 (46.6)	
12.5–50 miles	324 (32.6)	564 (29.2)		321 (32.3)	
50–250 miles	181 (18.2)	396 (20.5)		189 (19.0)	
>250 miles	22 (2.2)	41 (2.1)		20 (2.0)	
Missing	2 (0.2)	1 (0.1)		1 (0.1)	
High School Education			0.677		0.986
≥29%	265 (26.6)	548 (28.4)		267 (26.8)	
20–29 %	241 (24.2)	478 (24.8)		246 (24.7)	
14–20%	276 (27.8)	495 (25.6)		266 (26.8)	
<14%	205 (20.6)	390 (20.2)		209 (21.0)	
Missing	8 (0.8)	20 (1.0)		7 (0.7)	
Residence area			0.096		0.508
Metro	877 (88.1)	1648 (85.4)		866 (87.0)	
Urban	81 (8.2)	211 (10.9)		98 (9.9)	
Rural	11 (1.1)	27 (1.4)		8 (0.8)	
Missing	26 (2.6)	45 (2.3)		23 (2.3)	
Insurance			0.592		0.977
Not insured	32 (3.2)	67 (3.5)		35 (3.5)	
Private	328 (33.0)	694 (35.9)		342 (34.4)	
Medicaid	127 (12.8)	244 (12.6)		124 (12.5)	
Medicare	476 (47.8)	876 (45.4)		463 (46.5)	
Other	18 (1.8)	30 (1.6)		16 (1.6)	
Unknown	14 (1.4)	20 (1.0)		15 (1.5)	
Median household income, USD			0.863		0.987
<40,227	223 (22.4)	435 (22.5)		220 (22.1)	
40,227–50,353	200 (20.1)	405 (21.0)		206 (20.7)	
50,354–63,332	243 (24.4)	438 (22.7)		242 (24.3)	
>63,333	320 (32.2)	633 (32.8)		320 (32.2)	
Missing	9 (0.9)	20 (1.0)		7 (0.7)	
Tumor Stage			0.199		>0.99
Stage I	773 (77.7)	1458 (75.5)		774 (77.8)	
Stage II	222 (22.3)	473 (24.5)		221 (22.2)	
Tumor size, cm			0.144		0.568
<2	246 (24.7)	424 (22.0)		247 (24.8)	
2–3	332 (33.4)	634 (32.8)		352 (35.4)	
3–5	417 (41.9)	873 (45.2)		396 (39.8)	
Surgery			<0.001		0.888
Wedge/Segmentectomy	811 (81.5)	1356 (70.2)		811 (81.5)	
Lobectomy	143 (14.4)	415 (21.5)		148 (14.9)	
Extended Lobectomy	10 (1.0)	74 (3.8)		7 (0.7)	
Hepatectomy (NOS)	31 (3.1)	86 (4.5)		29 (2.9)	

Data are given as No. (%) unless otherwise noted. Pearson χ^2^ or Fisher’s exact test was performed between the study groups dependent on sample size. Percentages have been rounded and may not total 100. Abbreviations: MILR, minimally invasive liver resection; OLR, open liver resection; USD, US dollar. ^1^ MILR vs. Unmatched OLR, ^2^ MILR vs. Matched OLR.

**Table 2:** Pathological and Early Postoperative Outcomes of Patients with Hepatocellular Carcinoma in the Matched Data Set.

Variable	MILR	OLR	*p*
	*n* = 995	*n* = 995	
Tumor stage			0.261
pT0/pT1	631 (63.4)	650 (65.3)	
pT2	308 (31.0)	288 (28.9)	
pT3	6 (0.6)	1 (0.1)	
pT4	5 (0.5)	4 (0.4)	
Not available	45 (4.5)	52 (5.2)	
Nodal stage			<0.001
pN0	380 (38.2)	305 (30.7)	
pN1	4 (0.4)	1 (0.1)	
pNx	467 (46.9)	530 (53.3)	
Not available	144 (14.5)	159 (16.0)	
Lymph-Vascular invasion			0.853
No	667 (67.0)	675 (67.8)	
Yes	178 (17.9)	179 (18.0)	
Unknown	150 (15.1)	141 (14.2)	
Resection margin			0.006
Negative	891 (89.5)	927 (93.2)	
Positive	69 (6.9)	38 (3.8)	
Unknown	35 (3.5)	30 (3.0)	
Hospital stay, median (IQR), days	4 (3–6)	5 (4–8)	<0.001
Unplanned readmission within 30 days	40 (4.0)	54 (5.4)	0.169
30-day mortality	33 (3.3)	45 (4.5)	0.204
90-day mortality	45 (4.5)	67 (6.7)	0.041

Data are given as No. (%) unless otherwise noted. Pearson χ^2^ or Fisher’s exact test was performed for categorical parameters dependent on sample size. Wilcoxon rank sum test was performed for continuous parameters. Percentages have been rounded and may not total 100.Abbreviations: MILR, minimally invasive liver resection; OLR, open liver resection; NOS, not otherwise specified; IQR, interquartile range.

## Data Availability

This study used de-identified data from the National Cancer Data Base. The Commission on Cancer and the American College of Surgeons have not verified the data and are not responsible for analytic or statistical methodology employed or the conclusions drawn from this study.

## Supplementary Materials

The following are available online at https://www.mdpi.com/article/10.3390/cancers13194800/s1, Figure S1: Covariate balance measured by the standardized mean difference in the unmatched and matched cohort, Table S1: Univariate and multivariate logistic regression analysis of clinical factors associated with a positive resection margin in the matched data set, Table S2: Univariate and multivariate logistic regression analysis of clinical factors associated with 90-day mortality in the matched data set, Table S3: Predictors of death (overall survival) of patients with hepatocellular carcinoma in the matched data set.

## References

[1] Sung H., Ferlay J., Siegel R.L., Laversanne M., Soerjomataram I., Jemal A., Bray F. (2021). Global Cancer Statistics 2020: GLOBOCAN Estimates of Incidence and Mortality Worldwide for 36 Cancers in 185 Countries. CA Cancer J. Clin..

[2] Gordan J.D., Kennedy E.B., Abou-Alfa G.K., Beg M.S., Brower S.T., Gade T.P., Goff L., Gupta S., Guy J., Harris W.P. (2020). Systemic Therapy for Advanced Hepatocellular Carcinoma: ASCO Guideline. J. Clin. Oncol..

[3] Forner A., Reig M., Bruix J. (2018). Hepatocellular carcinoma. Lancet.

[4] Llovet J.M., Kelley R.K., Villanueva A., Singal A.G., Pikarsky E., Roayaie S., Lencioni R., Koike K., Zucman-Rossi J., Finn R.S. (2021). Hepatocellular carcinoma. Nat. Rev. Dis. Primers.

[5] Sapisochin G., Bruix J. (2017). Liver transplantation for hepatocellular carcinoma: Outcomes and novel surgical approaches. Nat. Rev. Gastroenterol. Hepatol..

[6] Di Sandro S., Benuzzi L., Lauterio A., Botta F., De Carlis R., Najjar M., Centonze L., Danieli M., Pezzoli I., Rampoldi A. (2019). Single Hepatocellular Carcinoma approached by curative-intent treatment: A propensity score analysis comparing radiofrequency ablation and liver resection. Eur. J. Surg. Oncol..

[7] Vitale A., Trevisani F., Farinati F., Cillo U. (2020). Treatment of Hepatocellular Carcinoma in the Precision Medicine Era: From Treatment Stage Migration to Therapeutic Hierarchy. Hepatology.

[8] Vogel A., Martinelli E., ESMO Guidelines Committee (2021). Updated treatment recommendations for hepatocellular carcinoma (HCC) from the ESMO Clinical Practice Guidelines. Ann. Oncol..

[9] Reich H., McGlynn F., DeCaprio J., Budin R. (1991). Laparoscopic excision of benign liver lesions. Obs. Gynecol..

[10] Wakabayashi G., Cherqui D., Geller D.A., Buell J.F., Kaneko H., Han H.S., Asbun H., O’Rourke N., Tanabe M., Koffron A.J. (2015). Recommendations for laparoscopic liver resection: A report from the second international consensus conference held in Morioka. Ann. Surg..

[11] Abu Hilal M., Aldrighetti L., Dagher I., Edwin B., Troisi R.I., Alikhanov R., Aroori S., Belli G., Besselink M., Briceno J. (2018). The Southampton Consensus Guidelines for Laparoscopic Liver Surgery: From Indication to Implementation. Ann. Surg..

[12] El-Gendi A., El-Shafei M., El-Gendi S., Shawky A. (2018). Laparoscopic Versus Open Hepatic Resection for Solitary Hepatocellular Carcinoma Less Than 5 cm in Cirrhotic Patients: A Randomized Controlled Study. J. Laparoendosc. Adv. Surg. Tech. A.

[13] Winchester D.P., Stewart A.K., Phillips J.L., Ward E.E. (2010). The national cancer data base: Past, present, and future. Ann. Surg. Oncol.

[14] Von Elm E., Altman D.G., Egger M., Pocock S.J., Gotzsche P.C., Vandenbroucke J.P., Initiative S. (2007). The Strengthening the Reporting of Observational Studies in Epidemiology (STROBE) statement: Guidelines for reporting observational studies. PLoS Med..

[15] Mokdad A.A., Minter R.M., Zhu H., Augustine M.M., Porembka M.R., Wang S.C., Yopp A.C., Mansour J.C., Choti M.A., Polanco P.M. (2017). Neoadjuvant Therapy Followed by Resection Versus Upfront Resection for Resectable Pancreatic Cancer: A Propensity Score Matched Analysis. J. Clin. Oncol..

[16] Rajyaguru D.J., Borgert A.J., Smith A.L., Thomes R.M., Conway P.D., Halfdanarson T.R., Truty M.J., Kurup A.N., Go R.S. (2018). Radiofrequency Ablation Versus Stereotactic Body Radiotherapy for Localized Hepatocellular Carcinoma in Nonsurgically Managed Patients: Analysis of the National Cancer Database. J. Clin. Oncol..

[17] Austin P.C. (2014). The use of propensity score methods with survival or time-to-event outcomes: Reporting measures of effect similar to those used in randomized experiments. Stat. Med..

[18] Benson A.B., D’Angelica M.I., Abbott D.E., Abrams T.A., Alberts S.R., Saenz D.A., Are C., Brown D.B., Chang D.T., Covey A.M. (2017). NCCN Guidelines Insights: Hepatobiliary Cancers, Version 1.2017. J. Natl. Compr. Cancer Netw..

[19] Fretland A.A., Dagenborg V.J., Bjornelv G.M.W., Kazaryan A.M., Kristiansen R., Fagerland M.W., Hausken J., Tonnessen T.I., Abildgaard A., Barkhatov L. (2018). Laparoscopic Versus Open Resection for Colorectal Liver Metastases: The OSLO-COMET Randomized Controlled Trial. Ann. Surg..

[20] Pinnock C.A., Haden R.M., Pinnock C., Lin T., Smith T. (2009). The surgical insult. Fundamentals of Anaesthesia.

[21] Buunen M., Gholghesaei M., Veldkamp R., Meijer D.W., Bonjer H.J., Bouvy N.D. (2004). Stress response to laparoscopic surgery: A review. Surg. Endosc..

[22] Cheung T.T., Dai W.C., Tsang S.H., Chan A.C., Chok K.S., Chan S.C., Lo C.M. (2016). Pure Laparoscopic Hepatectomy Versus Open Hepatectomy for Hepatocellular Carcinoma in 110 Patients with Liver Cirrhosis: A Propensity Analysis at a Single Center. Ann. Surg..

[23] Fu X.T., Tang Z., Chen J.F., Shi Y.H., Liu W.R., Gao Q., Ding G.Y., Song K., Wang X.Y., Zhou J. (2021). Laparoscopic hepatectomy enhances recovery for small hepatocellular carcinoma with liver cirrhosis by postoperative inflammatory response attenuation: A propensity score matching analysis with a conventional open approach. Surg. Endosc..

[24] Yoon Y.I., Kim K.H., Kang S.H., Kim W.J., Shin M.H., Lee S.K., Jung D.H., Park G.C., Ahn C.S., Moon D.B. (2017). Pure Laparoscopic Versus Open Right Hepatectomy for Hepatocellular Carcinoma in Patients with Cirrhosis: A Propensity Score Matched Analysis. Ann. Surg..

[25] Eguia E., Sweigert P.J., Li R.D., Kuo P.C., Janjua H., Abood G., Baker M.S. (2021). Laparoscopic partial hepatectomy is cost-effective when performed in high volume centers: A five state analysis. Am. J. Surg..

[26] Van der Poel M.J., Besselink M.G., Cipriani F., Armstrong T., Takhar A.S., van Dieren S., Primrose J.N., Pearce N.W., Abu Hilal M. (2016). Outcome and Learning Curve in 159 Consecutive Patients Undergoing Total Laparoscopic Hemihepatectomy. JAMA Surg..

[27] Donadon M., Terrone A., Procopio F., Cimino M., Palmisano A., Vigano L., Del Fabbro D., Di Tommaso L., Torzilli G. (2019). Is R1 vascular hepatectomy for hepatocellular carcinoma oncologically adequate? Analysis of 327 consecutive patients. Surgery.

[28] Yeh C.N., Lee W.C., Jeng L.B., Chen M.F. (2002). Pedunculated hepatocellular carcinoma: Clinicopathologic study of 18 surgically resected cases. World J. Surg..

[29] Vergara V., Muratore A., Bouzari H., Polastri R., Ferrero A., Galatola G., Capussotti L. (2000). Spontaneous rupture of hepatocelluar carcinoma: Surgical resection and long-term survival. Eur. J. Surg. Oncol..

[30] Ruzzenente A., Bagante F., Ratti F., Alaimo L., Marques H.P., Silva S., Soubrane O., Endo I., Sahara K., Beal E.W. (2020). Minimally Invasive Versus Open Liver Resection for Hepatocellular Carcinoma in the Setting of Portal Vein Hypertension: Results of an International Multi-institutional Analysis. Ann. Surg. Oncol..

[31] Syn N.L., Kabir T., Koh Y.X., Tan H.L., Wang L.Z., Chin B.Z., Wee I., Teo J.Y., Tai B.C., Goh B.K.P. (2020). Survival Advantage of Laparoscopic Versus Open Resection For Colorectal Liver Metastases: A Meta-analysis of Individual Patient Data from Randomized Trials and Propensity-score Matched Studies. Ann. Surg..

