# Supplementary material for: Minimally Invasive versus Open Liver Resection for Stage I/II Hepatocellular Carcinoma
Source: Cancers (Basel). 2021 Sep 25;13(19):4800. doi: 10.3390/cancers13194800 (PMC8507639; doi:10.3390/cancers13194800)
Supplement: Supplementary file 1 [file cancers-13-04800-s001.zip › cancers-1362491-supplementary.pdf]

Supplemental Data

Table S1: Univariate and multivariate logistic regression analysis clinical factors associated with a positive resection margin in the matched data set

	**Univariate Analysis**			**Multivariate Analysis**		
**Variable**	**OR**	**95% CI**	**P**	**OR**	**95% CI**	**P**
Approach						
OLR	Reference					
MILR	1.88	1.25-2.82	0.002	1.89	1.25-2.85	0.002
Age, years						
≤ 49	Reference					
50-59	2.00	0.69-5.76	0.199			
60-70	1.61	0.57-4.57	0.373			
≥ 65	1.91	0.66-5.51	0.234			
Sex						
Male	Reference					
Female	0.81	0.52-1.26	0.349			
Race						
White	Reference					
Black	0.72	0.39-1.31	0.281			
Other/Unknown	NA					
Comorbidity Index						
0	Reference					
1	1.15	0.75-1.76	0.535	1.11	0.72-1.73	0.627
2	0.28	0.07-1-12	0.080	0.26	0.06-1.06	0.062
3	0.79	0.39-1-63	0.528	0.76	0.37-1.56	0.453
Year of diagnosis						
2010	Reference					
2011	1.41	0.62-3.23	0.416			
2012	0.79	0.31-2.00	0.627			
2013	1.56	0.71-3.45	0.269			

2014	1.19	0.53-2.66	0.675			
2015	1.21	0.56-2.62	0.625			
Facility Location						
New England	Reference					
Middle Atlantic	1.19	0.53-2.66	0.674	1.35	0.59-3.07	0.473
South Atlantic	0.67	0.28-1.62	0.371	0.72	0.30-1.77	0.483
East North Central	0.89	0.38-2.09	0.784	0.93	0.39-2.22	0.871
East South Central	0.44	0.11-1.68	0.228	0.49	0.13-1.92	0.305
West North Central	0.23	0.05-1.11	0.067	0.26	0.06-1.26	0.094
West South Central	0.77	0.29-2.05	0.599	0.87	0.32-2.34	0.780
Mountain	1.42	0.45-4.55	0.551	1.46	0.45-4.76	0.527
Pacific	1.13	0.47-2.71	0.787	1.15	0.47-2.80	0.761
Missing	0.64	0.08-5.36	0.681	0.48	0.05-4.10	0.499
Facility type						
Non-Academic	Reference					
Academic	0.64	0.43-0.97	0.033	0.61	0.40-0.94	0.025
Surgery						
Wedge/Segmentectomy	Reference					
Lobectomy	0.57	0.29-1.10	0.092	0.60	0.31-1.18	0.139
Extended Lobectomy	NA			NA		
Hepatectomy (NOS)	0.27	0.04-1.97	0.196	0.29	0.04-2.16	0.228
Tumor Stage						
Stage I	Reference					
Stage II	1.39	0.90-2.14	0.141			
Tumor size, cm						
< 2	Reference					
2-3	0.87	0.54-1.41	0.575			
3-5	0.67	0.41-1.09	0.106			

Abbreviations: OR, odds ratio, MILR; minimally invasive liver resection; OLR, open liver resection

Factors associated with a p-value ≤ 0.100 were included in a multivariate analysis

Table S2: Univariate and multivariate logistic regression analysis of clinical factors associated with 90-day mortality in the matched data set

	**Univariate Analysis**			**Multivariate Analysis**		
	**OR**	**95% CI**		**OR**	**95% CI**	
**Variable**			**P**			**P**
Approach						
OLR	Reference					
MILR	0.66	0.45-0.97	0.033	0.65	0.44-0.96	0.032
Age, years						
≤ 49	Reference					
50-59	2.87	0.87-9.49	0.084	3.52	1.04-11.90	0.043
60-70	2.44	0.75-7.97	0.139	2.83	0.85-9.49	0.091
≥ 65	2.47	0.74-8.25	0.141	2.39	0.70-8.20	0.168
Sex						
Male	Reference					
Female	0.92	0.60-1.41	0.707			
Race						
White	Reference					
Black	0.64	0.35-1.18	0.156			
Other/Unknown	1.78	0.53-5.96	0.351			
Comorbidity Index						
0	Reference					
1	1.37	0.89-2.12	0.158	1.49	0.95-2.35	0.082
2	0.83	0.33-2.13	0.704	0.91	0.35-2.38	0.852
3	1.93	1.11-3.37	0.021	1.98	1.11-3.52	0.020
Year of diagnosis						
2010	Reference					
2011	0.96	0.49-1.89	0.905	0.93	0.47-1.87	0.840
2012	0.39	0.17-0.89	0.026	0.33	0.14-0.77	0.010
2013	0.61	0.30-1.24	0.176	0.52	0.25-1.08	0.078
2014	0.58	0.29-1.16	0.121	0.55	0.27-1.12	0.098
2015	0.73	0.39-1.37	0.328	0.67	0.35-1.29	0.230
Facility Location						

New England	Reference					
Middle Atlantic	0.81	0.35-1.88	0.629			
South Atlantic	0.95	0.41-2.21	0.909			
East North Central	0.68	0.28-1.66	0.400			
East South Central	1.90	0.75-4.84	0.177			
West North Central	0.35	0.09-1.35	0.127			
West South Central	1.42	0.59-3.43	0.433			
Mountain	0.54	0.11-2.62	0.444			
Pacific	0.98	0.40-2.40	0.962			
Missing	0.64	0.08-5.36	0.681			
Facility type						
Non-Academic	Reference					
Academic	0.61	0.41-0.91	0.015	0.58	0.38-0.88	0.011
Surgery						
Wedge/Segmentectomy	Reference					
Lobectomy	3.12	2.04-4.80	<0.001	3.11	1.99-4.84	<0.001
Extended Lobectomy	3.05	0.68-13.60	0.140	3.84	0.83-17.90	0.083
Hepatectomy (NOS)	3.02	1.32-6.89	0.008	3.97	1.69-9.32	0.002
Tumor Stage						
Stage I	Reference					
Stage II	1.43	0.93-2.18	0.100	1.42	0.92-2.21	0.111
Tumor size, cm						
< 2	Reference					
2-3	1.63	0.94-2.84	0.084	1.43	0.81-2.53	0.220
3-5	1.67	0.97-2.86	0.062	1.41	0.81-2.47	0.229

Abbreviations: OR, odds ratio; MILR, minimally invasive liver resection; OLR, open liver resection; NA, not available; NOS, not otherwise specified
Factors associated with a p-value ≤ 0.100 were included in a multivariate analysis

Table S3: Predictors of death (overall survival) of patients with hepatocellular carcinoma in the matched data set

Variable	Adjusted HR*	95% CI	P
Surgical approach			
OLR	Reference		
MILR	1.02	0.88-1.20	0.763
Surgery			
Wedge/Segmentectomy	Reference		
Lobectomy	1.29	1.04-1.59	0.020
Extended Lobectomy	1.07	0.44-2.62	0.880
Hepatectomy (NOS)	1.14	0.70-1.86	0.589
Tumor Stage			
Stage I	Reference		
Stage II	1.47	1.23-1.75	<0.001
Tumor size, cm			
< 2	Reference		
2-3	1.21	0.97-1.51	0.090
3-5	1.38	1.12-1.71	0.003
Grading			
Well-differentiated	Reference		
Moderate-differentiated	1.19	0.98-1.46	0.094
Poorly differentiated/undifferentiated	1.71	1.33-2.18	<0.001
Not determined	1.31	0.97-1.80	0.090
Lymph-Vascular invasion			
No	Reference		
Yes	1.42	1.16-1.74	<0.001
Unknown	1.25	0.99-1.56	0.055
Resection status			
Negative	Reference		
Positive	2.25	1.70-2.99	<0.001
Unknown	1.37	0.95-1.98	0.092

* Adjustment of patient and facility factors were accounted in this model (age, sex, race, facility location, facility type, facility distance, education, insurance status, comorbidity index, year of diagnosis, area of living, household income)

Abbreviations: HR, hazard ratio; MILR, minimally invasive liver resection; OLR, open liver resection

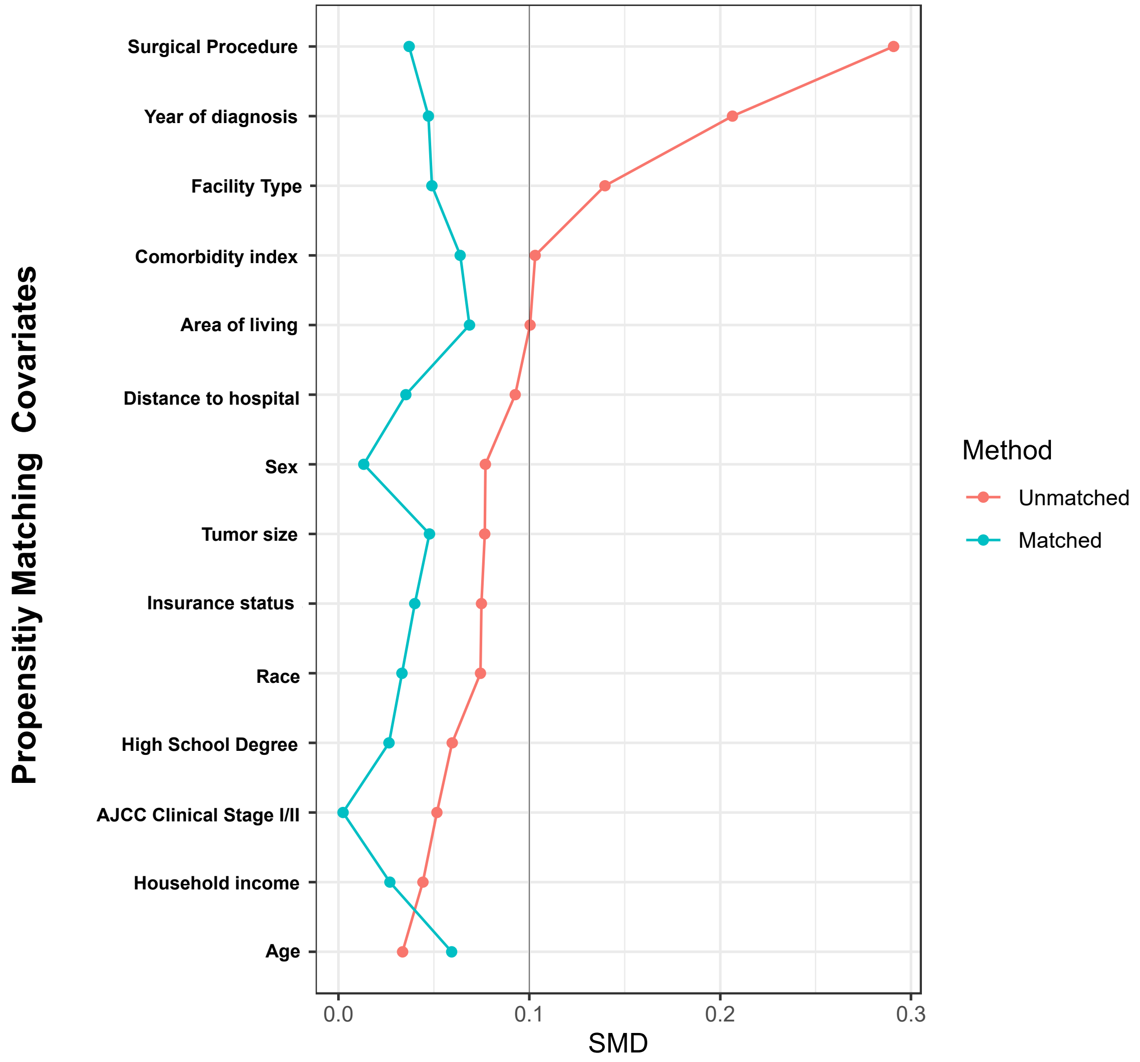

Figure S1: Covariate balance measured by the standardized mean difference in the Unmatched and Matched Cohort
